# The psychosocial impact of cancer diagnosis in pregnancy: a multicenter cohort study of psychosocial challenges in pregnant cancer patients and their partners

**DOI:** 10.3389/fpsyg.2025.1696459

**Published:** 2026-01-14

**Authors:** Evangeline A. Huis in 't Veld, Mathilde van Gerwen, Elisabeth M. van Dijk-Lokkart, Tineke Vandenbroucke, Rana Dandis, Charlotte L. LeJeune, Indra van Assche, Elyce Cardonick, Inge T. A. Peters, Anna Fagotti, Christianne Lok, Ingrid Boere, Marjon A. de Boer, Britt B. M. Suelmann, Sanne J. Gordijn, Nelleke Ottevanger, Mina Mhallem, Ruud Bekkers, Philippe Tummers, Daniela Chieffo, Martine van Grotel, Kristel Van Calsteren, Marry M. van den Heuvel-Eibrink, Frédéric Amant

**Affiliations:** 1 Center for Gynecological Oncology Amsterdam, Antoni van Leeuwenhoek-Netherlands Cancer Institute, Amsterdam, Netherlands; 2 Princess Máxima Center for Pediatric Oncology, Utrecht, Netherlands; 3 Cancer Center Amsterdam, Research Program, Amsterdam, Netherlands; 4 Department of Child & Adolescent Psychiatry and Psychosocial Care, Emma Children's Hospital, Amsterdam UMC, University of Amsterdam, Amsterdam, Netherlands; 5 Amsterdam Reproduction and Development, Child Development, Amsterdam, Netherlands; 6 Multidisciplinary Breast Center, UZ Leuven, Leuven, Belgium; 7 Department of Obstetrics and Gynaecology, Division of Oncology, UZ Leuven, Leuven, Belgium; 8 Laboratory of Gynaecological Oncology, Department of Oncology, KU Leuven, Leuven, Belgium; 9 Unit of Woman and Child, Department of Development and Regeneration, KU Leuven, Leuven, Belgium; 10 Department of Obstetrics and Gynecology, Cooper University Health Care, Camden, NJ, United States; 11 Gynecologic Oncology Unit, Department of Woman's and Child Health and Public Health Sciences, Fondazione Policlinico Universitario Agostino Gemelli IRCCS, Rome, Italy; 12 Catholic University of the Sacred Heart, Rome, Italy; 13 Department of Medical Oncology, Erasmus MC Cancer Center, Rotterdam, Netherlands; 14 Department of Obstetrics and Gynaecology, Amsterdam UMC, Vrije Universiteit Amsterdam, Amsterdam, Netherlands; 15 Amsterdam Reproduction & Development Research Institute, Amsterdam, Netherlands; 16 Department of Medical Oncology, UMC Utrecht, Utrecht, Netherlands; 17 Department of Obstetrics, University Medical Center Groningen, University of Groningen, Groningen, Netherlands; 18 Medical Oncology, Radboud University Medical Center, Nijmegen, Netherlands; 19 Department of Obstetrics, Cliniques Universitaires Saint-Luc, Brussels, Belgium; 20 Department of Gynecology, Catharina Hospital, Eindhoven, Netherlands; 21 Grow, School for Oncology and Reproduction, Maastricht University, Maastricht, Netherlands; 22 Department of Gynecologic Oncology, Radboud UMC, Nijmegen, Netherlands; 23 Department of Human Structure and Repair, Gynecology, Gent University Hospital, Ghent, Belgium; 24 Clinical Psychology Unit, Fondazione Policlinico Universitario Agostino Gemelli IRCCS, Rome, Italy; 25 UMCU-Wilhelmina Children's Hospital, Utrecht, Netherlands; 26 Unit of Gynaecological Oncology, Department of Oncology, KU Leuven, Leuven, Belgium; 27 Department of Obstetrics and Gynaecology, Division of Gynaecological Oncology, UZ Leuven, Leuven, Belgium

**Keywords:** antenatal cancer, cognitive coping, distress, partners, patients, questionnaires

## Abstract

**Objective:**

Cancer during pregnancy poses significant physical and psychological challenges for expectant mothers and their partners. While elevated stress and anxiety levels in pregnancy are well-documented, the specific parental psychological impact of cancer during pregnancy remains unexplored, and could be different for patients and partners. This study represents the first full prospective analysis, complementing earlier retrospective research by analyzing self-reported perspectives and coping strategies among both pregnant cancer patients and their partners.

**Methods:**

Participants were prospectively recruited within the International Network on Cancer, Infertility, and Pregnancy (INCIP) across The Netherlands, Belgium, USA and Italy. Participants completed the Cancer and Pregnancy Questionnaire (CPQ), developed by our research team, and a shortened version of the Cognitive Emotion Regulation Questionnaire (CERQ). Differences in concerns and coping strategies were analyzed using Wilcoxon Signed-Rank tests, Cliff's Delta for effect sizes, and Spearman's correlation analysis.

**Results:**

Between 2009 and 2024, 220 patients and 189 partners participated. Compared to partners, patients reported significantly greater concerns about their child's health and the implications of the disease, treatment, and upcoming delivery. Patients exhibited higher use of both adaptive (e.g., Positive Reappraisal, Refocusing) and maladaptive (e.g., Self-blame, Rumination) coping strategies, and a stronger desire to continue the pregnancy. Satisfaction with medical care was similar between groups. Maternal age at diagnosis was linked to maladaptive strategies (Catastrophizing and Blaming Others), while higher gestational age at diagnosis was associated with both adaptive (Positive Refocusing and Positive Reappraisal), and the maladaptive coping strategy Catastrophizing.

**Conclusions:**

This study provides valuable insights into the psychological challenges experienced by pregnant cancer patients and their partners, emphasizing the need for personalized care that includes psychological support from the start.

## Background

A cancer diagnosis during pregnancy, occurs in approximately 1 in every 1,000 pregnancies, and presents profound medical and psychological challenges (Ma et al., 2022). Advances in oncology, maternal-fetal medicine and offspring research have led to feasible and safe treatment protocols in pregnancy that consider the health needs of both mother and fetus (Maggen et al., 2019; Amant et al., 2012; Van Assche et al., 2023; Vandenbroucke et al., 2020; Amant et al., 2019; Huis In 't Veld et al., 2025). Pregnancy itself is inherently a stressful life event, which induces (hormonal related) emotional fluctuations and, in general accompanied by uncertainty regarding the transition to parenthood. The added burden of a cancer diagnosis, which is also characterized by significant emotional distress about personal health, mortality and the well-being of the child, is likely to further exacerbate this vulnerability (Harrison, 2013). Although several studies have examined the elevated levels of anxiety and stress in healthy pregnant women, research on the combined burden of the combination of a cancer diagnosis and pregnancy remains limited (Nielsen-Scott et al., 2022; Cena et al., 2021; Van den Bergh et al., 2020; Henry et al., 2012).

A previously published meta-analysis reported that 15% of pregnant women in the general population are diagnosed with an anxiety disorder (Dennis et al., 2017) which can result in poor maternal health and adverse birth outcomes (Hadfield et al., 2022). For pregnant (breast)cancer patients, additional concerns include the impact of treatment on their unborn child, their estimated prognosis, the ability to breastfeed, and the long-term impact for both the mothers and her baby (Harris et al., 2023; Ives et al., 2012; Henry et al., 2012). Anxiety and concerns during pregnancy are not limited to only the pregnant women themselves. For partners in the general population, the prevalence of anxiety during the perinatal period has been reported to be around 10% (Rao et al., 2020), which is important to consider, as not only maternal health but also the mental health of partners may impact pregnancy outcomes (Thiel et al., 2020; Wickersham et al., 2020).

A previous mixed retrospective-prospective study shows that coping strategies may play a key role in how pregnant patients and their partners manage the intertwined challenges, showing that adaptive strategies, such as acceptance and positive reappraisal, are associated with lower levels of anxiety and depression, while maladaptive strategies, including rumination, and self-blame may increase distress (Vandenbroucke et al., 2017). However, existing literature focusing specifically on the psychological impact of cancer during pregnancy is mainly based on retrospective studies which may be affected by recall bias and show considerable variability in the measurement and reporting of distress levels and coping strategies, making it difficult to accurately determine these issues in this population (Harris et al., 2023). Furthermore, these studies often do not distinguish between the experiences of mothers and partners, despite research on other disorders revealing significant sex differences in the psychosocial experiences of parents (Clarke et al., 2009).

Gaining deeper insight into the potential differences between pregnant cancer patients and their partners regarding the emotional and psychological impact of cancer during pregnancy and their coping strategies is relevant for improving patient care. This study represents the first prospective analysis of the psychological impact of cancer during pregnancy, and aims to complement earlier retrospective research by analyzing self-reported perspectives on the pregnancy complicated by cancer and by determining coping strategies of both pregnant cancer patients and their partners.

## Methods

### Study participants

Pregnant cancer patients and their partners were prospectively identified from the international registry of the International Network on Cancer, Infertility and Pregnancy (INCIP) prior to delivery and invited to participate in this study, which was conducted in The Netherlands, Belgium, the United States, and Italy. Just after cancer diagnosis, all participants were asked to complete study questionnaires. Ethical approval was obtained by all participating centers and written informed consent was collected from all participating patients, and their partners. The study has been registered with ClinicalTrials.gov (NCT00330447).

### Measures

Maternal oncological and obstetrical data were collected from medical records. All patients and their partners were asked to complete the Cancer and Pregnancy Questionnaire (CPQ), developed within our research team, which measures concerns and perspectives about the cancer diagnosis during pregnancy. Patients filled out the questionnaire from their perspective regarding their diagnosis, while partners completed the same questionnaire from their perspective about their partners' diagnosis. The questionnaire had a two-part structure, with the first part consisting of 5 subscales with a total of 40 items: (1) concerns regarding the (unborn) child's health; (2) concerns about the maternal disease and treatment; (3) concerns about the pregnancy and delivery; (4) satisfaction with the information and care provided by the medical team, and (5) the tendency to maintain the pregnancy. The full questionnaire and details on its design by the INCIP is available in the Supporting Information (S1-S2). Participants indicated how well the statements reflected their thoughts using a Likert scale, ranging from 1 (“*not at all*”) to 7 (“*very well*”), and 8 (“*not applicable*”). As this questionnaire was developed as an exploratory research instrument, it does not include cut off scores, and responses are interpreted descriptively rather than diagnostically. Responses are interpreted descriptively rather than diagnostically.

The second part of the questionnaire consisted of 27 items, and was based on the internationally validated Cognitive Emotion Regulation Questionnaire (CERQ), developed to identify one's cognitive emotion regulation (or cognitive coping) strategies after having experienced a negative and stressful event (Garnefski et al., 2001). Consistent with a previous study by INCIP, we opted to use the validated shortened version of the CERQ, with 3 items per subscale, asking the participants to respond how they feel, using a Likert scale, ranging from 1 (“*(almost) never*”) to 5 (“(*almost*) *always*”) (Vandenbroucke et al., 2017). The subscales assess nine specific cognitive coping strategies, categorized into adaptive and maladaptive coping styles, as shown in Supporting Information S3. Adaptive coping styles include Acceptance, Positive Refocusing, Refocusing on Planning, Positive Reappraisal, and Putting into Perspective. Maladaptive coping strategies include Self-blame, Rumination, Catastrophizing, and Blaming Others. Similarly to the CPQ, this short form of the CERQ does not include clinical or diagnostic cut-off values. Consistent with its original validation, subscale scores represent the relative frequency with which a cognitive coping strategy is used and are not intended to classify individuals into normative or clinical categories.

### Inclusion and exclusion criteria

Inclusion criteria were: (1) a diagnosis of cancer during an ongoing pregnancy; (2) enrolment within the INCIP registry prior to delivery; and (3) ability to provide written informed consent. Partners of eligible patients were also invited to participate. Exclusion criteria were: insufficient language proficiency to complete study questionnaires, cognitive impairment inhibiting informed participation, severe clinical deterioration preventing questionnaire completion, or absence of an ongoing pregnancy at the time of recruitment. These criteria are consistent with the INCIP study protocol and aim to ensure reliable self-report data.

### Statistical analyses

For the first part of the questionnaire which measured the patients and partners' perspectives across five categories, responses marked as “8” (not applicable) on the Likert scale were excluded from the analysis to avoid potential bias, as these responses did not reflect participants‘ thoughts directly. For analysis, we first grouped responses into categories as outlined in the Supporting Information S1, separately for patients and partners. Next, we calculated the mean score per category as outcome measure. Given the non-normal distribution of the data, we analyzed the median scores for each category as the primary outcome measure for reported concerns.

A Spearman's correlation analysis was conducted to examine the associations between concerns and cancer type, as well as between coping styles and cancer type. Furthermore, potential relationships between experienced concerns and coping styles were explored for both patients and partners.

To enable comparison between patients and partners, we performed Wilcoxon Signed-Rank tests to determine whether differences in perspectives regarding the pregnancy complicated by a cancer diagnosis were statistically significant between the two groups. To further evaluate the magnitude and clinical relevance of these differences, we calculated Cliff's Delta (δ) as a measure of effect size, which represents the proportion of all possible patient-partner response pairs in which the patient reported a higher concern than partners, minus the proportion in which the opposite was true (Cliff, 1996). Effect sizes were interpreted as follows: δ > 0.33 (large effect), 0.147 ≤ δ ≤ 0.33 (moderate effect), and δ < 0.147 (small effect). This combined approach allowed us to identify not only statistically significant differences but also those that may have clinical significance. The same approach was used to assess differences in coping styles between patients and partners.

## Results

### Participant characteristics

A total of 220 women and 189 partners completed the questionnaires between 2009 and 2024. Eighteen (8%) women were previously described by our group as part of another study (Vandenbroucke et al., 2017). Median age of the patients at diagnosis was 33 years (Inter Quartile Range (IQR) 30–36) and the median age of partners at time of completing the questionnaire was 35 years (IQR 32–39). Information on the sex of the partner was not available. Gestational age at diagnosis was 33 weeks (IQR 13.64–23.75).

A total of 116 patients (53%) was multiparous, and 74 patients (34%) were nulliparous at time of cancer diagnosis (median parity 1, IQR 1.00–2.00). For the remaining 30 (13%) patients, data regarding their gravidity, parity or reproductive status at the time of cancer diagnosis was not available. Table 1 summarizes all participant characteristics including disease- and pregnancy-related characteristics. Although 220 pregnant patients completed the questionnaires, only 189 partners participated. Not all women had an available partner at the time of diagnosis, and some partners declined participation, were not reachable, or did not complete the questionnaire despite initial enrolment. A minority of women were not in a relationship at the time of diagnosis.

**Table 1:** Demographic characteristics of the patients.

**Characteristic**	**All patients (*N* = 220)^a^**
Cancer during pregnancy—number (%)	
Breast cancer	112 (50.9)
Hematological cancer	30 (13.6)
Hodgkin Lymphoma	17 (56.6)
Non-Hodgkin Lymphoma	9 (30)
B-Cell Lymphoma	1 (3.3)
Follicular Lymphoma	1 (3.3)
Acute Lymphatic Leucemia (ALL)	1 (3.3)
Chronic Lymphatic Leucemia (CLL) phenotype B	1 (3.3)
Cervical cancer	23 (10.5)
Ovarian cancer	17 (7.7)
Skin cancer	9 (4.1)
Thyroid cancer	6 (2.7)
Brain cancer	2 (0.9)
Bone tumors	2 (0.9)
Bladder cancer	1 (0.5)
Endometrial cancer	1 (0.5)
Other	6 (2.7)
Unknown	21 (9.5)
Maternal age at diagnosis—years	33 (30–36)
Gestational age at diagnosis—weeks	17 (13.64–23.75)
Age partner at completion questionnaire—years	35 (32.00–39.00)
Gravida	2 (1.00–3.00)
Number of living children at diagnosis	1 (0.00–1.25)
*^a^* n (%); Median (IQR) IQR = Inter Quartile Range

### Exploration of potential risk factors for measured perspectives and used coping strategies

Spearman's correlation analysis showed no significant associations between the five categories measuring the perspectives and maternal cancer type (all *p* > 0.05). Similarly, no significant associations were found between measured perspectives and gestational age at diagnosis nor between the measured perspectives and the year of diagnosis (all *p* > 0.05). However, when associations were explored between the measured perspectives and maternal age at diagnosis, the Spearman's rank correlation showed a significant association (ρ = 0.11, *p* = 0.03).

When the same associations were explored with the used coping strategies, no significant associations were found with maternal cancer type (all *p* > 0.05). However, the Spearman's rank correlation showed a significant association with maternal age at diagnosis for the maladaptive coping strategies Catastrophizing (ρ = −0.07, *p* < 0.001) and Blaming Others (ρ = 0.06, *p* = 0.02). Also, significant associations were found between gestational age at diagnosis and the adaptive coping strategies Positive Refocusing (ρ = −0.07, *p* = 0.02), Positive Reappraisal (ρ = −0.12, *p* < 0.001), Putting Perspective (ρ = −0.09, *p* < 0.001), and with the maladaptive strategy Catastrophizing (ρ = −0.08, *p* < 0.001).

Additionally, associations were explored between the used coping strategies and year of diagnosis, revealing a significant association with the adaptive coping strategies Acceptance (ρ = −0.08, *p* < 0.001), Positive Refocusing and the maladaptive coping strategy Catastrophizing (ρ = −0.09, *p* < 0.001). However, a t-test comparing differences in measured perspectives between patients diagnosed in earlier (2009–2015) versus later (2016–2020) years did not reveal any significant differences (all *p* > 0.05).

Finally, Spearman's correlation analysis did not reveal any significant relationship between the various perspectives and coping styles.

### Comparing patients and their partner's perspectives about the cancer diagnosis

**Figure 1:**
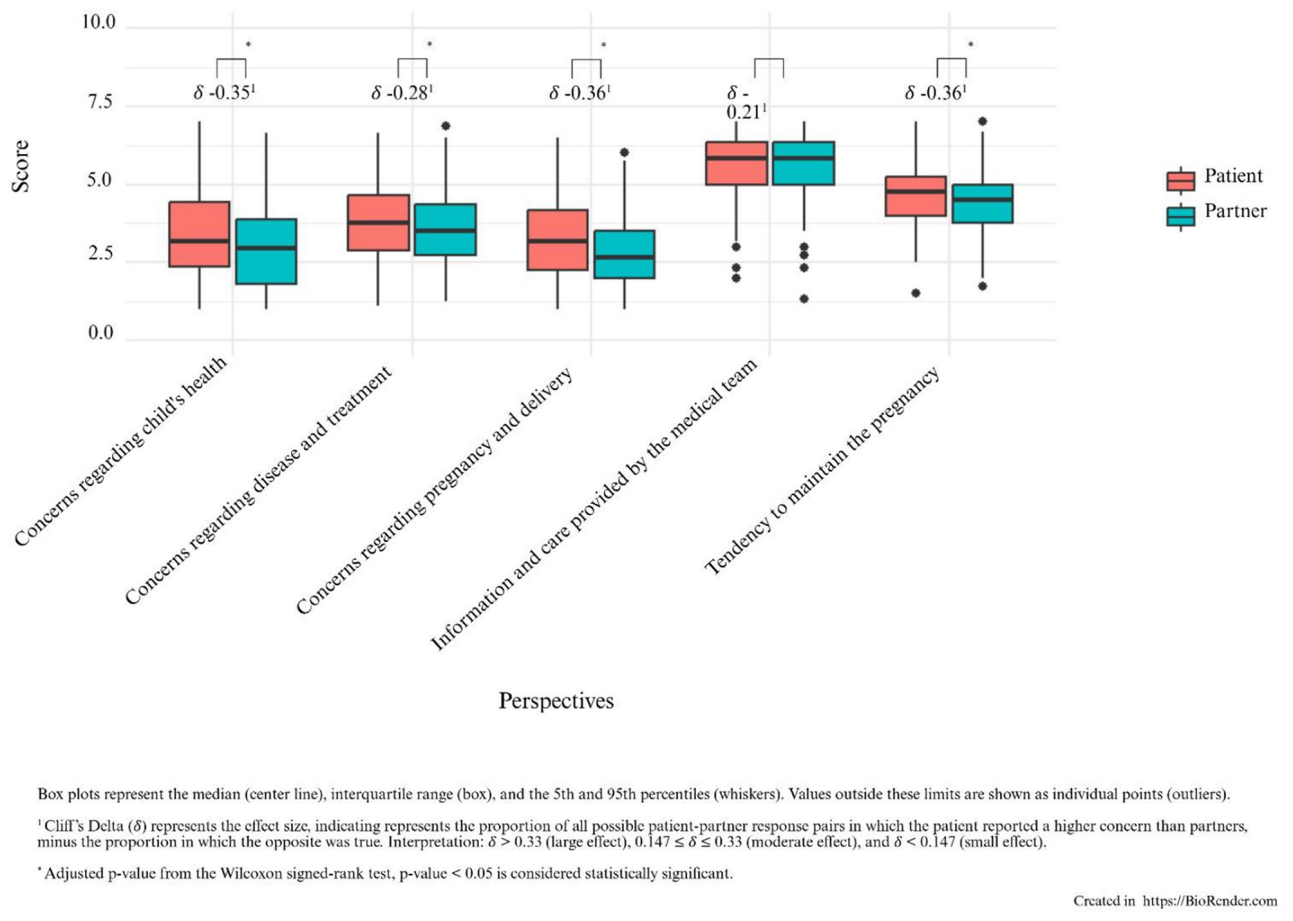
Differences in perspectives regarding pregnancy and treatment for patients and partners.

The boxplots in Figure 1 display the comparison of scores between patients and partners across the five categories of the CPQ, including Cliff's Delta (δ) as a measure of effect size.

Although on average, pregnant cancer patients score around the mid-range on the CPQ, the results show that they significantly experienced more concerns than their partners on both the (unborn) child's health (*W* =16577, *p* < 0.001) and regarding disease, treatment (*W* =18136, *p* = 0.05), pregnancy and upcoming delivery (*W* = 16180, *p* < 0.001). Both patients and partners reported comparable levels of satisfaction with the information and care provided (*W* = 20113, *p* = 0.81). Finally, the wish to continue their pregnancy was generally somewhat stronger among patients than their partners (*W* = 16203, *p* < 0.001).

### Comparing cognitive coping strategies of patients, and partners

**Figure 2:**
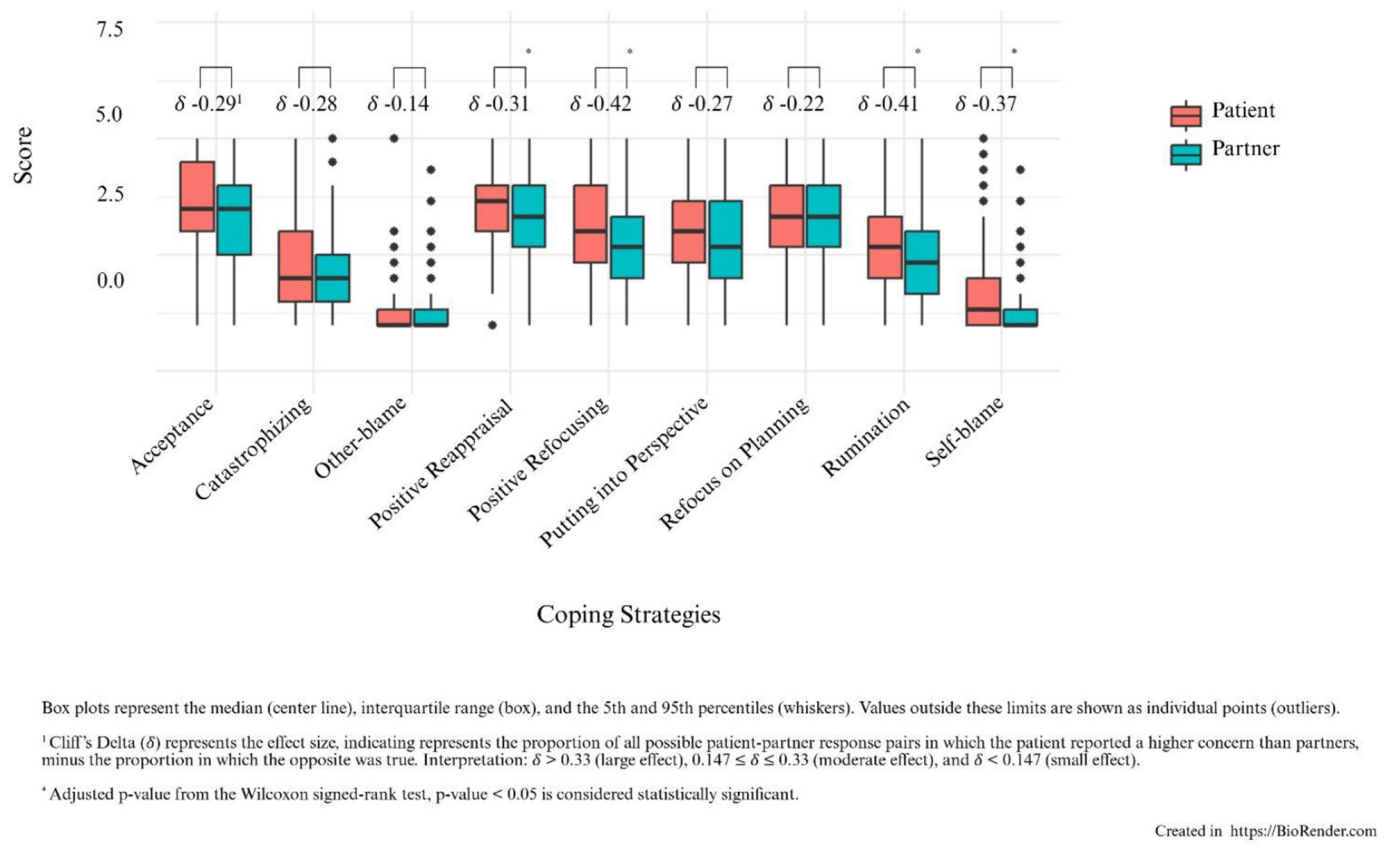
Differences in coping strategies for patients and partners.

The boxplots in Figure 2 display the comparison of the used coping categories between patients and partners as measured by the CERQ, including Cliff's Delta (δ) as a measure of effect size.

Overall, the results indicate that patients tend to use certain coping strategies more frequently than their partners, both adaptive and maladaptive. Specifically, patients reported significantly greater use of the adaptive coping strategies Positive Reappraisal (*W* = 17382, *p* = 0.01) and Positive Refocusing (*W* = 14820, *p* < 0.001). Regarding maladaptive coping, patients showed significantly higher levels of Rumination (*W* = 14976, *p* < 0.001) and Self-blame (*W* = 15891, *p* < 0.001), compared to their partners.

## Discussion

In this multicenter study, 220 pregnant cancer patients and 189 partners completed self-reported questionnaires on their perspectives on the pregnancy complicated by cancer and used coping strategies during pregnancy. This study represents the first fully prospective analysis in this domain, complementing earlier retrospective research and laying the fundament for future research. Our findings suggest potential predictors for perspectives on pregnancy and disease, as well as the coping strategies used by pregnant cancer patients and their partners. Specifically, we found that a higher maternal age at diagnosis was associated with greater concerns about disease and treatment. This relationship was weak but statistically significant. Additionally, older pregnant cancer patients appeared less likely to engage in the coping strategy Catastrophizing, but were more inclined to Blame Others. In contrast, women diagnosed later in pregnancy were less likely to use adaptive coping strategies such as Positive Refocusing, Positive Reappraisal, and Putting things into Perspective, while also being less prone to use the maladaptive coping strategy Catastrophizing. Lastly, although the associations were weak, pregnant cancer patients that were diagnosed in more recent years were less likely to accept the situation, use the adaptive coping strategy Positively Refocus, and were less likely to Catastrophize.

Furthermore, the comparison of patients and partners reveal that patients reported significantly greater concerns on all the domains compared to partners, suggesting a heightened emotional burden during pregnancy. This is in line with previous research in the general population, reporting that expecting mothers tend to experience more stress and anxiety, compared to partners (Darwin et al., 2017). This anxiety can be in part be caused by worries about the unborn child's health, as similar patterns have been observed in parents of children with medical conditions. However, in these studies both parents report higher levels of anxiety, impacting their and their child's quality of life (Boettcher et al., 2021; Clarke et al., 2009).

Our findings further suggest that while patients may be more proactive in seeking adaptive coping mechanisms (such as Positive Refocusing and Reappraisal) compared to their partners, they also may need additional psychological support to address the maladaptive tendencies they tend to use (Kozu et al., 2020).

While both patients and partners are satisfied with the information received from the medical team, their perspectives diverge when it comes to concerns about the (unborn) child's health, and the direct physical and emotional experiences related to the disease, treatment, pregnancy, and childbirth. It remains unclear whether this pattern is attributed to the direct physical and emotional involvement of the patient in the pregnancy or to the combination with a cancer diagnosis. This also emphasized that a personalized approach for support during cancer in pregnancy is warranted. Previous research, however, highlights that also healthy women, particularly those experiencing their first pregnancy, often describe childbirth as an anxious event, largely due to feelings of uncertainty and lack of control until the baby is born (McCarthy and Houghton, 2021). This uncertainty is even more pronounced in our cohort, where illness adds an additional layer of unpredictability.

Furthermore, our results show that patients were more inclined to continue their pregnancy, compared to partners, despite facing significant concerns regarding the cancer diagnosis and the subsequent pregnancy-related challenges. This is consistent with previous studies describing that, although there were no statistically significant differences in experienced concerns between pregnant cancer patients and their partners (in contrast with our results), patients were still more likely to choose to continue the pregnancy (Vandenbroucke et al., 2017).

Despite these significant differences in concerns, the raw reported levels of concerns were not particularly high, generally ranging from 3 to 4 on a 7-point Likert scale. This may be due to the increasing knowledge in this area, combined with the fact that all patients in our study were seen by specialists and healthcare providers who are experts in this field. The ongoing child follow-up study from the INCIP, which has assessed offspring up to 15 years post-delivery, provides reassuring data regarding child development, which is incorporated into patient counseling at diagnosis (Amant et al., 2012, 2015; Van Assche et al., 2024; Vandenbroucke et al., 2020; Van Gerwen et al., 2021; Van Assche et al., 2023; Huis In 't Veld et al., 2025). Although the current study did not find significant differences in parental concerns between patients diagnosed in earlier (2009–2015) versus later (2016–2020) years, patients diagnosed more recently did show a greater tendency to use positive coping strategies such as Acceptance and Positive Refocusing, and were less likely to use the maladaptive coping strategy Catastrophizing. This may suggest that access to long-term follow-up information and improved counseling over time contribute to a higher level of reassurance and understanding among both patients and partners. As a result, the increasing knowledge and the information provided to patients may play a role in their overall satisfaction with the care received, suggesting that effective counseling and support may help mitigate anxiety. This highlights the potential benefits of centralizing care for these patients within a multidisciplinary team of experts, similar to the advice of the Advisory Board on Cancer, Infertility and Pregnancy (Amant et al., 2022; Heimovaara et al., 2022) and similar to how pediatric follow-up care for the offspring of cancer patients already has been centralized in The Netherlands. Moreover, while the role of partner support in our cohort remains unclear, previous research in the general population suggests that practical and emotional support from a partner plays a crucial role in reducing stress levels (Hoffmann et al., 2023). Given that in our population, the woman is the one facing illness, it is conceivable that partners take on a significant supportive role. Actively exploring and strengthening this support system through psychological counseling may be a key element of comprehensive patient care, helping to improve coping strategies and overall well-being. The current study highlight the importance of recognizing the partner's role not only in decision-making, but also in emotional support, which may enhance dyadic coping and improve overall psychological well-being (Traa et al., 2015).

## Clinical implications

Patient support organizations, such as the Dutch *Stichting Ster(k)* or the British Mummy's Star can play a key role in the process of comprehensive information provision and offering peer support, reducing feelings of isolation among pregnant cancer patients. Furthermore, specialized outpatient services, such as the Cancer in Pregnancy Offspring outpatient Clinic in the Princess Máxima Center in The Netherlands provide follow-up screening and counseling postpartum, when new challenges arise. By combining personalized advice and interventions, peer support, and continuous follow-up, healthcare systems may be able to provide more integrated care that addresses both the psychological and medical needs of patients and their families during the perinatal and postnatal period.

## Study strengths and limitations

A major strength from our study is that it is based on data from a unique international collaboration, allowing for a broader perspective on the psychological impact of cancer during pregnancy. Our study also has some limitations. There may be a bias in participant attrition, as those that were very ill, or diagnosed as terminal may have dropped out, while patients who were more engaged in their care or had higher health literacy were more likely to enroll, which may limit the generalizability of our findings. Due to the prospective nature of the study, (obstetric) treatment plans and estimated prognoses were not always known at the moment of data collection, making it impossible to assess the potential confounding impact of illness severity. Moreover, as the study was cross-sectional rather than longitudinal, we were unable to assess how anxiety or coping may fluctuate over the course of pregnancy in response to factors such as test results, fetal growth, or treatment response. A key limitation of this study is the absence of standardized mental-health outcomes such as anxiety, depression, or distress scales. As these were not part of the INCIP protocol, we were unable to formally assess the psychological consequences associated with coping patterns. The lack of normative data for the study instruments further limits the ability to make direct comparisons. Furthermore, it remains important to consider the diversity within the study population, as detailed information is lacking about impact factors such as educational background, religion, estimated prognosis and stage of disease, gravidity, pre-existing psychiatric disorders, and the use of antidepressants, as well as other demographic variables which are likely to influence perceptions of care and measured concerns. Future research should aim to recruit larger patient populations and include validated mental-health instruments with cut-off points to allow a more comprehensive understanding of psychological functioning.

## Conclusion

This study provides further insight into the psychological concerns and coping strategies of both pregnant patients and their partners following a cancer diagnosis, building upon the previous study conducted by the INCIP (Vandenbroucke et al., 2017). Given the significant emotional burden, it may be helpful that a psychologist is included as part of the multidisciplinary care team from the start. This could involve using screening tools and structured conversations that take personal factors such as maternal age and gestational age at diagnosis into account, ensuring that both patients and their partners receive personalized care that addresses their specific needs. This approach could help them to navigate this impactful life period and cope as effectively as possible. While these results offer new insights, they mark only the beginning of understanding the psychological challenges associated with cancer during pregnancy. Moving forward, future research may focus on identifying the most effective screening tools for psychological distress and further exploring best practices for providing individualized support to improve the overall well-being of patients and their families.

## Data Availability

The original contributions presented in the study are included in the article/supplementary material, further inquiries can be directed to the corresponding author.
